# Saliva of *Rhipicephalus* (*Boophilus*) *microplus* (Acari: Ixodidae) inhibits classical and alternative complement pathways

**DOI:** 10.1186/s13071-016-1726-8

**Published:** 2016-08-11

**Authors:** Naylene C. S. Silva, Vladimir F. Vale, Paula F. Franco, Nelder F. Gontijo, Jesus G. Valenzuela, Marcos H. Pereira, Mauricio R. V. Sant’Anna, Daniel S. Rodrigues, Walter S. Lima, Blima Fux, Ricardo N. Araujo

**Affiliations:** 1 Departamento de Parasitologia, Laboratório de Fisiologia de Insetos Hematófagos, Universidade Federal de Minas Gerais, Belo Horizonte, MG Brazil; 2 Laboratório de Simulídeos e Oncocercose, Instituto Oswaldo Cruz, FIOCRUZ, Rio de Janeiro, RJ Brazil; 3 Instituto Nacional de Ciência e Tecnologia em Entomologia Molecular, Rio de Janeiro, 21941-591 Brazil; 4 Vector Molecular Biology Section, LMVR, National Institute of Allergy and Infectious Diseases, NIH, Rockville, MD USA; 5 Empresa de Pesquisa Agropecuária de Minas Gerais, Fazenda Experimental Santa Rita, Rodovia MG 424 km 64, Caixa Postal 295, Prudente de Morais, 35701-970 MG Brazil; 6 Departamento de Patologia, Universidade Federal do Espírito Santo, Vitória, MG Brazil

**Keywords:** *Rhipicephalus* (*Boophilus*) *microplus*, Saliva, Complement inhibition, Classical pathway, Alternative pathway

## Abstract

**Background:**

*Rhipicephalus* (*Boophilus*) *microplus* is the main ectoparasite affecting livestock worldwide. For a successful parasitism, ticks need to evade several immune responses of their hosts, including the activation of the complement system. In spite of the importance of *R. microplus*, previous work only identified one salivary molecule that blocks the complement system. The current study describes complement inhibitory activities induced by *R. microplus* salivary components and mechanisms elicited by putative salivary proteins on both classical and alternative complement pathways.

**Results:**

We found that *R. microplus* saliva from fully- and partially engorged females was able to inhibit both pathways. Saliva acts strongly at the initial steps of both complement activation pathways. In the classical pathway, the saliva blocked C4 cleavage, and hence, deposition of C4b on the activation surface, suggesting that the inhibition occurs at some point between C1q and C4. In the alternative pathway, saliva acts by binding to initial components of the cascade (C3b and properdin) thereby preventing the C3 convertase formation and reducing C3b production and deposition as well as cleavage of factor B. Saliva has no effect on formation or decay of the C6 to C8 components of the membrane attack complex.

**Conclusion:**

The saliva of *R. microplus* is able to inhibit the early steps of classical and alternative pathways of the complement system. Saliva acts by blocking C4 cleavage and deposition of C4b on the classical pathway activation surface and, in the alternative pathway, saliva bind to initial components of the cascade (C3b and properdin) thereby preventing the C3 convertase formation and the production and deposition of additional C3b.

**Electronic supplementary material:**

The online version of this article (doi:10.1186/s13071-016-1726-8) contains supplementary material, which is available to authorized users.

## Background

The cattle tick *Rhipicephalus* (*Boophilus*) *microplus* is the primary ectoparasite affecting livestock worldwide leading to considerable economic losses for cattle farming in tropical and subtropical areas of world [1–3]. This tick species also transmits pathogens, such as *Babesia* spp. and *Anaplasma* spp., that affect cattle farming [4].

While feeding, ticks, including *R. microplus*, secrete an arsenal of bioactive molecules that modulate the host immune system and haemostasis, which aids in the haematophagy and in the transmission of pathogens [5–7]. Indeed, ticks have salivary molecules that are capable of inhibiting the complement system [8–11]. Since the first description of complement inhibition by tick saliva in *Ixodes dammini* (syn. of *I. scapularis*, see [12]) [13], several tick salivary anti-complement molecules have been described [10, 14–16]. In spite of its importance, to date only one work has described the action of *R. microplus* saliva on the complement system [17].

The complement system is one the first lines of defence from vertebrate hosts and can be triggered by three pathways: the classical, the lectin and the alternative pathways [18]. The classical pathway is triggered when C1q binds to the Fc region of antigen-bound IgM or IgG antibodies. Each C1rC1s dimer, composed of one C1r and one C1s subunit, binds to one C1q to form a C1 complex, which cleaves C4 and C2 to give C4b and C2a. These attach to the activation surface to form the C4bC2a enzyme (otherwise known as C3 convertase of the classical pathway). The C3 convertase cleaves multiple C3 molecules into C3b and C3a. C3b then associates with C4b from the C3 convertase to form the C5 convertase of the classical pathway (C4bC2aC3b) [19, 20].

The lectin pathway shares components with the classical pathway, but instead of antibodies and C1, the cascade is initiated by the binding of mannose-binding lectins (MBL) or ficolins to highly glycosylated pathogen-associated molecular patterns (PAMPs) present on the surface of a wide range of pathogens [21, 22].

Differently from the classical pathway, the alternative pathway is constantly activated and proceeds unless inactivated by plasma or membrane-bound regulatory molecules. The process is triggered when C3b, which is always present in small amounts in the serum and extracellular fluids from C3 hydrolysis, covalently binds to activation surfaces and to fB that is subsequently cleaved by factor D to give the C3 convertases of the alternative pathway (C3bBb). These, in turn, produce additional C3b molecules thereby amplifying the cascade [20, 23]. The C3 convertase is a labile enzyme stabilized by the aggregation of one properdin molecule that increases the half-life of the complex by at least 10-fold [24] and the ability of surface-bound C3b to interact with fB [25]. When a second C3b binds to the C3bBb complex, the alternative pathway C5 convertase is assembled (C3bBbC3b) [19, 20].

The C5 convertase formed from the three complement system activation pathways cleaves C5 into C5a and C5b thereby triggering the assembly of the membrane attack complex (MAC). Thus, C5b rapidly associates with C6, C7 and C8, and C9 molecules to form the C5b6789 or MAC, which is assembled on the surface of pathogens, thereby forming pores on the lipid bilayer of membranes [18, 26].

Complement pathways are highly regulated via many control proteins and intrinsic enzymatic activities that act mainly by: direct inhibition of serine proteases, decay and destruction of convertases and control of the MAC [19]. In addition to pores on lipid membranes, the complement is also an effector of humoral immunity and inflammation. Organism- or antigen-bound C3b aids in opsonisation and phagocytosis by effector cells in a process mediated by complement receptors [27, 28]. In addition, C3a and C5a are strong anaphylatoxins that induce damage by recruiting and stimulating granulocytes to release tissue-degrading enzymes, pro-inflammatory mediators and reactive oxygen species [18, 29].

Most of the described tick salivary proteins affecting the host’s complement system come from tick species belonging to the genus *Ixodes.* In addition, most inhibit the complement alternative pathway by binding to properdin thus inducing the decay of the C3 convertase and also dissociating Bb from C3b [15, 16]. Besides inhibiting the alternative pathway, the Tick Salivary Lectin Pathway Inhibitor (TSLPI) of *I. scapularis* is also known to inhibit the lectin pathway by preventing the binding of MBL and ficolins to their ligands thus blocking the activation of the complement cascade [30]. Two other salivary proteins are known to inhibit the complement pathways: one (named AamAV422) described in *Amblyomma americanum*, which blocks the classical pathway [31], and another (named OmCI) in the argasid *Ornithodoros moubata* [32]. However, the inhibition mechanisms elicited by AamAV422 remain unexplored whereas it is known that OmCI binds to C5, thereby inhibiting both, the classical and alternative pathways [32]. Saliva of the neotropical tick *Amblyomma cajennense* was also shown to inhibit the classical pathway; however the inhibitor was not identified [33]. Recently, a new family of C5 inhibitors was identified with homolog sequences in the species *Rhipicephalus appendiculatus*, *R. microplus*, *Dermacentor andersoni* and *Hyalomma marginatum* [17]. Members of this family were shown to act similarly to OmCI, but binding to a different site at the C5 component.

Efforts towards finding the elusive salivary proteins of *R. microplus* affecting the host complement system and their mechanisms of action may be an important step to understand the relationship between parasite and host and to assist the identification of new vaccine targets against this ectoparasite. Indeed, conventional tick control is based on the expensive and labor-intensive treatment of animals with acaricides but their improper use has increased the incidence of acaricide-resistant ticks [34]. Here we confirmed the complement inhibitor activity in *R. microplus* saliva and investigated other mechanisms elicited by putative salivary proteins on the classical and alternative complement pathways.

## Methods

### Obtaining ticks and saliva

*Rhipicephalus microplus* ticks were collected from naturally infested adult crossbred cattle (Gir × Holstein) maintained in the Santa Rita Experimental Farm of the Agricultural Research Company of Minas Gerais (EPAMIG), located at Prudente de Morais, MG (19°28'55"S, 44°09'18"W). Fully- (> 4 mm) and partially (< 4 mm) engorged females were carefully detached from cattle skin, cleaned with distilled water and attached onto a surface with double-sided adhesive tapes. The saliva was obtained by injection of 3 to 6 μl pilocarpine in 2 % PBS (136.8 mM NaCl; 2.7 mM KCl, 4.76 mM Na_2_HPO_4_ and 1.76 mM KH_2_PO_4_) into the ventro-lateral region of the body [14]. Saliva was collected for two hours directly from the mouthparts of the ticks using a pipette tip followed by centrifugation (14,000× *g* for 5 min) and the supernatant was stored at -80 °C until use. Salivary total protein concentration was determined using bovine serum albumin as standard [35]. Briefly, three replicates of each aliquoted saliva were diluted 1:10 in 20 μl distilled water and added to 200 μl Bradford solution (0.1 % Coomassie Brilliant Blue G-250, 5 % ethanol and 8.5 % phosforic acid). After 5 min, the mixture was measured using a spectrophotometer (UV-1650PC Spectrophotometer, Shimadzu) at 595 nm.

### Classical pathway haemolysis assays

Classical pathway haemolytic assays were carried out using sheep erythrocytes according to Mendes-Souza et al. [36], modified from Whaley & North [37]. Fresh sheep blood suspension (500 μl), conserved in Alsever’s solution at a ratio of 1:1, was washed three times with 5 ml GHB-EDTA buffer (0.1 % gelatin, 5 mM Hepes, 145 mM NaCl and 10 mM EDTA, pH 7.4) followed by sensitisation of the erythrocytes with sheep anti-erythrocyte antibody (1:1,000). The erythrocytes were washed once with 5 ml of GHB-EDTA buffer and twice with 5 ml GHB^2+^ (GHB buffer supplemented with 0.15 mM CaCl_2_ and 0.5 mM MgCl_2_, pH 7.4) by centrifugation at 470× *g* for 5 min at 4 °C. Erythrocyte concentration was adjusted to 2 × 10^8^ cells/ml before each experiment.

The haemolytic assays were conducted in a final volume of 125 μl containing up to 25 μl of tick saliva or saline (15.4 mM NaCl in 100 ml) and 50 μl of normal human serum (NHS) diluted in GHB^2+^ buffer at a ratio of 1:60. Aliquots of the erythrocyte solution (50 μl) were added to the assays and the reaction was incubated at 37 °C for 30 min. After incubation, the reaction was stopped by the addition of 500 μl of ice-cold saline. Haemolysis was measured spectrophotometrically at 414 nm. All assays were carried out in duplicate with at least three biological replicates. For each experiment, three different controls (without saliva and with pilocarpine) were used as follows: total haemolysis (saline was substituted by distilled water); positive control (haemolysis caused by serum without any sample); and negative control (without NHS where only spontaneous haemolysis is present). NHS aliquots (stored at -80 °C) were submitted to one freezing and thawing cycle and had their concentration adjusted in order to generate at least 90 % of the total haemolysis. The results were expressed as percentage of haemolysis inhibition in relation to the positive control.

### Alternative pathway haemolysis assays

For the alternative pathway, unsensitised rabbit blood was used as activating surface [36, 37]. Aliquots of fresh rabbit blood (500 μl) conserved in Alsever’s solution in a 1:1 ratio were washed three times with 5 ml Mg-EGTA solution (5 mM Hepes, 145 mM NaCl, 100 mM EGTA, 7 mM MgCl_2_, 0,1 % gelatin, pH 7.4) by centrifugation at 470 *g* for 5 min at 4 °C. EGTA chelates the divalent cation Ca^2+^, but not Mg^2+^, thereby inhibiting the activation of the classical pathway and leaving only the alternate pathway active. The concentration of erythrocytes was adjusted to 2 × 10^8^ cells/ml as above. The haemolytic assays were conducted using 50 μl (1:10) NHS diluted in Mg-EGTA buffer solution. The other procedures were similar to that used for the classical pathway described above.

### Enzyme-linked immunosorbent assays for the classical pathway

The classical pathway assays were performed as described before [38]. Each well of a 96-well microplate (Costar®, code 9017) was sensitised with 2 μg of purified human IgG in 50 μl of carbonate/bicarbonate buffer (15 mM Na_2_CO_3_, 35 mM NaHCO_3_, pH 9.6) by overnight incubation in a humid chamber. IgG was purified from human serum by affinity chromatography using a Protein A-conjugated Sepharose bead column [39].

The sensitised wells were blocked with 200 μl of blocking solution 1 (3 % skimmed milk powder in 10 mM Tris, 140 mM NaCl, pH 7.4) followed by the addition of 200 μl of blocking solution 2 (blocking solution 1 supplemented with 5 mM CaCl_2_), both incubated for 30 min at room temperature under agitation (150 rpm). After blocking, the saliva and human serum (1:100) in 100 μl of sample buffer (5 mM Hepes, 145 mM NaCl, 2 mM CaCl_2_, 1 mM MgCl_2_, pH 7.4) were incubated at 37 °C for 30 min under agitation. Positive and negative controls (similarly to the haemolytic assays) were also included. The wells were washed three times with 200 μl of washing solution (blocking solution 2 with 0.1 % skimmed milk powder) at room temperature for 2 min under agitation.

The components deposited on the plate surface after the classical pathway activation were detected using 50 μl/well of specific antibodies diluted in antibody buffer (10 mM Hepes, 140 mM NaCl pH 7.4) incubated for 30 min under agitation. The antibodies used are as follows: goat anti-human C1q (Sigma: C3900), goat anti-human C5b (Sigma: C3402) and goat anti-human C9 (Sigma: A226) antibodies diluted at 1:2500; rabbit anti-human C3b (Sigma: C3402) and rabbit anti-human C4b (Sigma: C3402) diluted at 1:1000. These antibodies were diluted in antibody solution (10 mM Hepes, 140 mM NaCl pH 7.4) and incubated for 30 min (150 rpm). Each well was then washed three times.

Antibodies were detected by the addition of 50 μl/well of peroxidase-conjugated antibodies (rabbit anti-goat IgG (Sigma: A5420) diluted at a ratio of 1:2500 or mice anti-rabbit IgG (Sigma: A1949) diluted at 1:1500) in antibody solution. After washing, bound components were developed by addition of 200 μl of peroxidase substrate solution (1 mg/ml O-Phenylene-diamine (Sigma: P9029), 50 mM sodium citrate, 0.075 % H_2_O_2_ (Synth: P22330) pH 5.5). Absorbance at 450 nm was measured in a microplate reader (Versamax, Molecular Devices) every 30 s for 10 (kinetic method) at 37 °C. The data generated were converted to percentage of deposition according to the following equation: [(Vmax_450nm_sample - Vmax_450nm_ sample solution)/(Vmax_450nm_ NHS - Vmax._450nm_ sample solution)] × 100.

Positive controls were considered as 100 % deposition. The results were expressed as relative percentage inhibition by comparing the deposit levels in the experimental assays with the deposit levels obtained in the positive control experiments.

### Enzyme-linked immunosorbent assays for the alternative pathway

Assays for the alternative pathway were performed with the addition of 100 μl/well of 0.1 % agarose solution and incubation at 37 °C for 24 h [38]. Saliva and NHS (7:100 ratio) were diluted in up to 100 μl of HMEBN solution (5 mM Hepes, 7 mM MgCl_2_, 10 mM EGTA, 5 mg/ml BSA, 145 mM NaCl, pH 7.4) were incubated for 30 min at 37 °C under agitation. After incubation, the wells were washed three times with 200 μl of HMEBN for two min at room temperature and under agitation. The components deposited on the plate surface after the activation of the alternative pathway were detected using 50 μl/well of the following specific antibodies: rabbit anti-human C3b, goat anti-human Bb (Comp Tech: A235), goat anti-human properdin (Comp Tech: A239), goat anti-human C5b or goat anti-human C9. Antibodies were diluted at 1:1000 in HN solution (10 mM Hepes, 145 mM NaCl, 0.1 % BSA, pH 7.4) and incubated for 30 min at room temperature under agitation. After incubation, the wells were washed as described above and 50 μl of peroxidase-conjugated mice anti-rabbit IgG antibody or rabbit anti-goat IgG. Conjugates were diluted at a 1:1500 ratio in HN solution and incubated for 30 min under agitation at room temperature. The wells were washed again and 200 μl of peroxidase substrate solution were added. Absorbance at 450 nm was measured as above.

### Western blot analysis of complement factors cleavage

The cleavage of C4 in human serum was assessed by Western blot using the supernatants from the classical pathway haemolytic assays [40]. Sample aliquots of 25 μl (saline or 15 μg salivary protein) were used to perform the classical pathway haemolytic assays as above. Reactions at 0 and 30 min were centrifuged (1,700 *g* for 1 min) and 25 μl of the supernatant in reducing and denaturing buffer (10 % SDS and 5 % β-2-Mercaptoetanol) was applied on 10 % SDS-PAGE electrophoretic analysis of the protein content [41]. The proteins in the gels were transferred to nitrocelulose membranes (Immunobilon-NC, Millipore) and incubated with goat anti-human C4 (Comp Tech: A205) diluted using a 1:2000 ratio in PBS/tween-20 for 2 h under agitation. The secondary antibody (rabbit anti-goat IgG peroxidase-conjugated) was diluted at a ratio of 1:4000 in PBS. Band identification was performed using the DAB Peroxidase Substrate Kit (Vector Laboratories, Burlingame, USA).

The C4 cleavage was also evaluated using purified components [40]. In 0.5 ml tubes, 0.6 μg of C1s was combined to 3 μg of C4 and 15 μg of salivary proteins in 30 μl PBS total volume. Tubes containing C4 alone were used as negative control while tubes containing C4 and C1s without saliva were used as positive control. A tube containing C4 plus saliva was also used as control to evaluate possible C4 cleavage by salivary proteases. Samples were incubated for 2 h at 37 °C and the enzymatic activity of C1s was measured by the presence of a 84 kDa α’ C4 fragment by Western blot as described above.

To analyse C3 cleavage, alternative pathway haemolytic assays were performed and analysed by Western blot as above except for the antibodies used: goat anti-human C3 (Comp Tech: A213) diluted using a 1:2000 ratio followed by addition of peroxidase-conjugated rabbit anti-goat IgG diluted at a ratio of 1:4000. For B cleavage analysis, the samples were applied on a native-PAGE and the antibodies used were goat anti-human factor B (Calbiochem®: 401504) diluted 1:1000 and rabbit anti-goat IgG peroxidase-conjugated diluted at 1:2000 [15].

The cleavage of factor B was also evaluated using purified complement components [15]. The assay was carried out by combining 0.5 μg factor D with 2 μg fB, 2 μg C3b and 15 μg of salivary proteins in 30 μl PBS final volume supplemented with 5 mM MgCl_2_. Tubes containing only fB were used as negative control while tubes containing fB, C3b and factor D without saliva were used as positive control. A control containing fB plus saliva evaluated the possible fB cleavage by salivary proteases. Samples were incubated for 30 min at 37 °C. The enzymatic activity of the factor D on factor B was measured by Western blot visualising a 60 kDa Bb fragment, as described above.

### Quantification of C3bBP formation by ELISA

The C3 convertase precursor (C3bBP) was synthesised *in vitro* by combining C3b (Comp tech: A114), factor B (Comp tech: A135) and properdin (Comp: A139) (modified from [42]). The wells of 96-well microplates were coated with 50 μl/well of C3b in PBS and incubated for 12 h at 4 °C in a humid chamber. The C3b-bound wells were then blocked with 200 μl of PBS/Tween-20 solution containing 1 % BSA for 2 h at room temperature under agitation. Afterwards, the wells were washed three times with washing buffer (8.1 mM Na_2_HPO_4_, 1.8 mM NaH_2_PO_4_, 25 mM NaCl, 10 mM MgCl_2_, 0.05 % Tween-20, pH 7.4) for 3 min under agitation. After the washings, up to 50 μl saliva, factor B and properdin in binding buffer (8.8 mM Na_2_HP0_4_, 1.8 mm NaH_2_P0_4_, 0.05 % Tween-20, 75 mM NaCl, 10 mM MgCl_2_, 1 % BSA, pH 7.4) were added to the wells and incubated for 30 min at 37 °C under agitation followed by one washing cycle. An antibody mixture (50 μl), containing either anti-fB or anti-properdin antibody in antibody binding buffer (8.8 mM Na_2_HP0_4_, 1.8 mM NaH_2_P0_4_, 0.05 % tween-20, 25 mM NaCl, 10 mM MgCl_2_, 1 % BSA) at pH 7.4, was incubated on the wells for 30 min at room temperature under agitation. This step was followed by three washes as described above. A solution (50 μl/well) of rabbit anti-goat IgG peroxidase-conjugated at a ratio of 1:2000 in antibody solution was added to the wells and incubated for 30 min at room temperature under agitation. After the washes, components bound to the plate surface were developed with addition of 200 μl of developing solution. Deposition levels (%) were calculated using the following equation: [(OD_450nm_sample - OD_450_nm only C3b in wells)/(OD_450nm_sample without saliva - OD_450nm_ only C3b in wells)] × 100 and used for statistical analysis.

### Binding of *R. microplus* saliva to components of the complement alternative pathway

To analyse whether salivary molecules bind to components of the complement alternative pathway, 96-well microplates were coated with 10 μg of *R. microplus* salivary protein or 1 % BSA (negative controls) diluted in 50 μl of carbonate/bicarbonate buffer by overnight incubation in a moist chamber at 4 °C. The wells were then blocked with 200 μl of blocking solution (PBS with 1 % BSA) and incubated for 1 h at room temperature under agitation. After blocking, 50 μl of PBS containing 0.2 μg of each component of the alternative pathway (C3b, factor B, properdin and factor Bb) were incubated for 30 min at 37 °C under agitation. Lastly, the wells were washed twice with 200 μl PBS/tween-20 at room temperature for 2 min under agitation.

To detect plate-bound C3b, properdin, factor B and Bb, the wells were firstly incubated with primary antibodies (rabbit anti-human C3b, goat anti-human properdin, goat anti-human factor B and goat anti-human Bb, respectively) diluted using a 1:5000 ratio, followed by the addition of the secondary antibodies (peroxidase-conjugated mice anti-rabbit IgG antibody and peroxidase-conjugated rabbit anti-goat IgG antibody at a dilution ratio of 1:3000). The components bound to the plates were exposed by the addition of 200 μl of developing solution. The measurements and data analyses were performed as described above.

### ELISA for evaluation of the decay of factors of the complement alternative pathway

ELISA assays were modified from a previously described protocol [16]. 96-well microplates were coated with 0.1 % agarose at 37 °C for 24 h and were then incubated with NHS in HMEBN solution at 37 °C for 30 min for convertases formation. Wells were washed with HMEBN solution and then incubated with 15 μg of salivary protein in HMEBN for 30 min at 37 °C. After incubation, wells were again washed with HMEBN and the detection of microplate-bound components (C3b, Bb, Properdin, C5b or C9) was performed using primary specific antibodies (rabbit anti-human C3b, goat anti-human Bb, goat anti-human properdin, goat anti-human C5b or goat anti-human C9) followed by a secondary peroxidase-conjugated mice anti-rabbit IgG or rabbit anti-goat IgG, both diluted in HN solution and incubated for 30 min at room temperature. The development and percent deposition of factors on microplates were analysed as described above.

### Membrane attack complex (MAC) deposition assessment by haemolytic assay

Classical pathway haemolytic assays were carried out as described in section 2.2 but using C6 (Comp tech: A323), C7 (Comp tech: A324) or C8 (Comp tech: A325) depleted serum diluted at 1:10 in GHB^2+^ [37]. Tubes were then centrifuged at 480 *g* for three min at 4 °C and erythrocytes were re-suspended in 300 μl of GHB-EDTA solution and used in a second round of classical pathway haemolytic assay with NHS under the same conditions described on item 2.2.

On the first round, the complement system was activated and the components were deposited on the membrane of erythrocytes up to the point where the absence of the specific component under investigation halted the progress of the pathway, ultimately preventing the formation of the membrane attack complex (MAC). In the second round, the component present in the NHS (which was missing in the first round) resumes MAC formation (except if MAC inhibitors are present in the saliva) without a further activation of the classical pathway which is blocked by EDTA.

### Statistical analysis

Statistical analysis was performed using GraphPad Prism 5.0 software. All experiments were performed with at least three biological replicates in duplicate (haemolytic assays) or triplicate (deposition assays). The difference between groups was assessed using *t*-test (when comparing two groups) and ANOVA followed by Dunnett’s or Bonferroni *post-hoc* tests (when comparing more than two groups). A 95 % significance level (*P* < 0.05) was set as statistically significant.

## Results

### *Rhipicephalus microplus* saliva inhibits both the classical and the alternative pathway

**Fig. 1:**
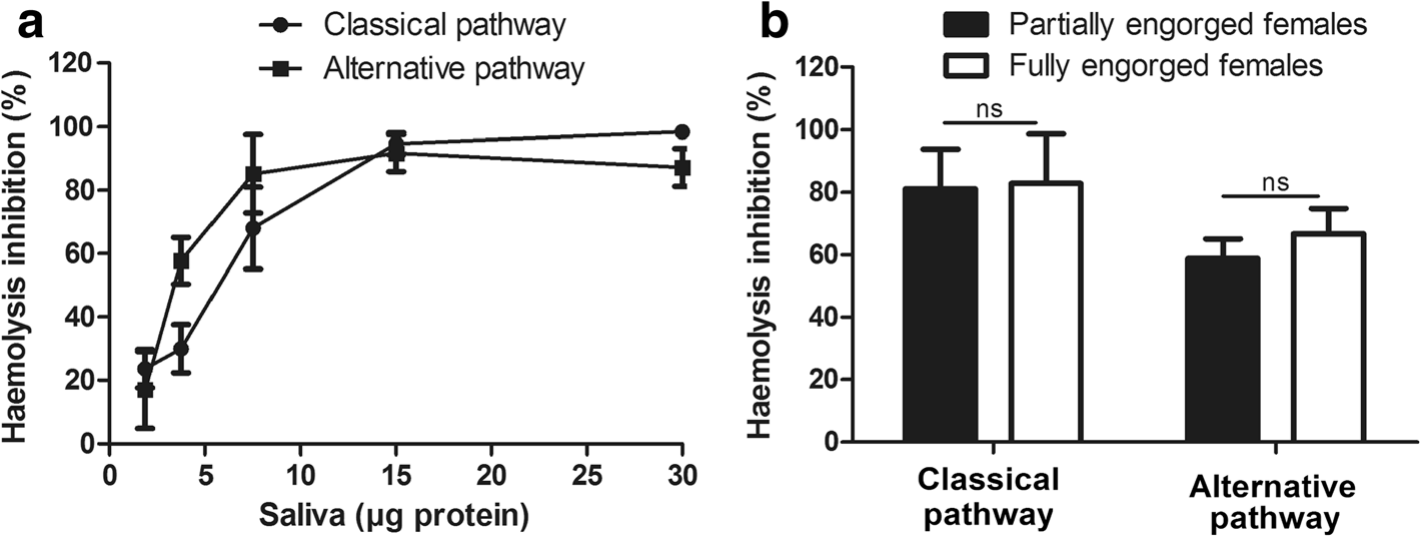
*Rhipicephalus microplus* salivary inhibition of haemolysis by the classical and alternative pathways. A total volume of 125 μl was used for the haemolytic assay. Human serum was added to assess the effects of saliva on the process of haemolysis via the classical (2.1 μl) and alternative (12.5 μl) complement pathways, inducing at least 90 % of haemolysis. In (**a)** different amounts of saliva were tested and in (**b)** 7.5 μg of salivary proteins was used. Bars represent the arithmetic mean ± standard deviation (SD) from three biological replicates. *Abbreviation*; ns, non-significant (*P* > 0.05)

Saliva from fully-engorged females was tested for inhibitory effects on the classical and alternative complement pathways using haemolytic assays. The results are summarized in Fig. 1a. *Rhipicephalus microplus* saliva was able to inhibit both of these pathways of the human complement system. This inhibition was dose-dependent: 5.09 μg was the amount of salivary proteins needed to inhibit 50 % of the classical pathway activity and 4.67 μg was the amount of salivary proteins needed to inhibit 50 % of the alternative pathway activity.

Fully- and partially engorged females produced different quantities and qualities of saliva when stimulated with pilocarpine. The average volumes and concentration of salivary proteins collected per tick were: 8.58 μl at 0.76 μg/μl (*n* = 55) and 1.52 μl at 1.89 μg/μl (*n* = 57) from fully- and partially engorged females, respectively. However, saliva from both engorgement phases produced a similar inhibitory effect on the classical (*t*-test: *t*_(4)_ = 1.27, *P* = 0.27) and alternative pathways (*t*-test: *t*_(4)_ = 0.19, *P* = 0.43) (Fig. 1b). As pilocarpine was used to stimulate tick salivation, pilocarpine control assays were carried out and showed no effect on either the classical or alternative pathways at 15, 30, 60 and 120 μg (Additional file 1: Figure S1).

### *Rhipicephalus microplus* saliva inhibits the deposition of C4 by the classical pathway

**Fig. 2:**
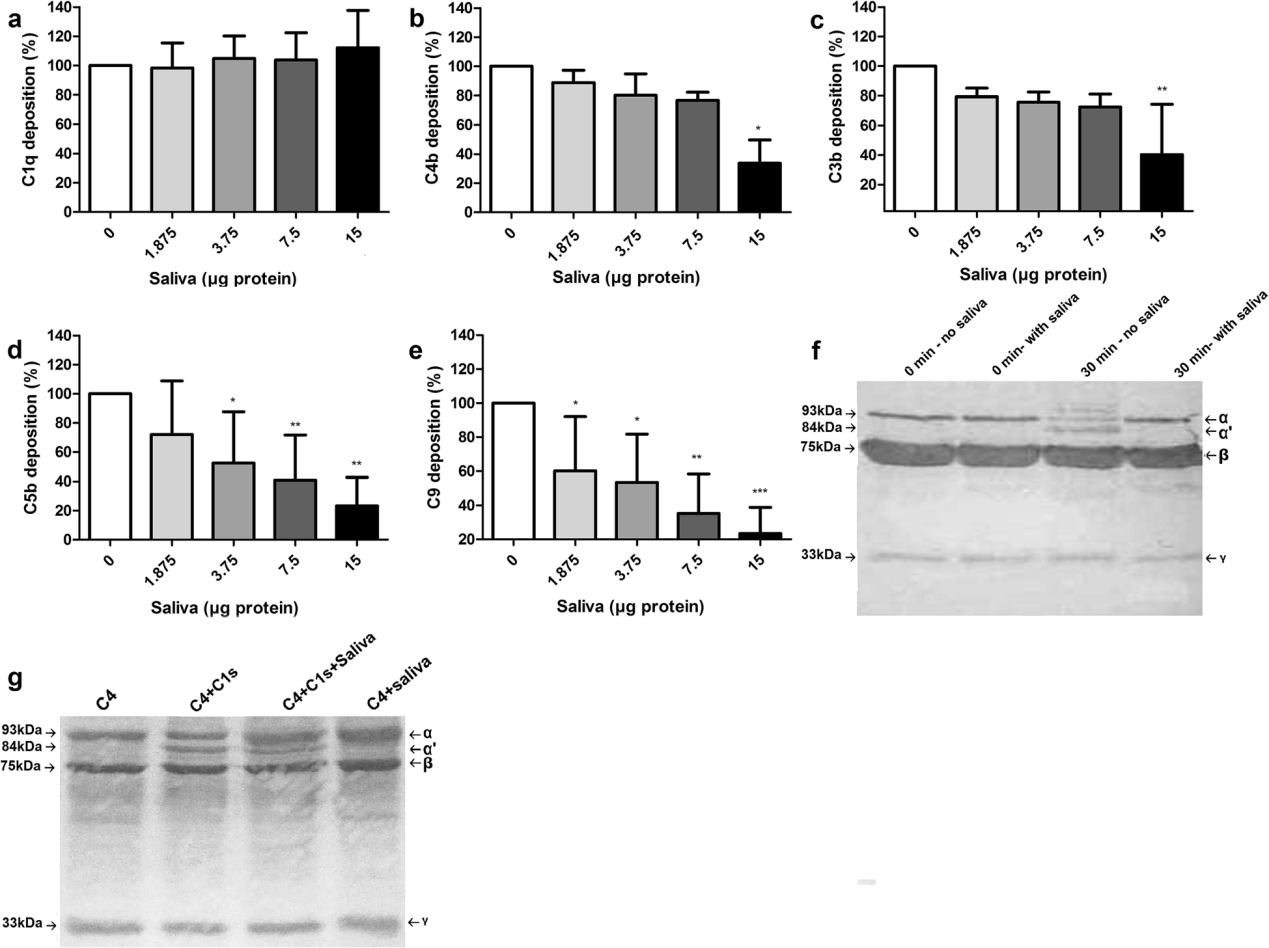
Effect of *Rhipicephalus microplus* saliva on the deposition and cleavage of components of the classical pathway. **a**-**e** Deposition of C1q, C4b, C3b, C5b and C9, respectively, on the activation surfaces. Bars represent the arithmetic mean ± standard deviation (SD) of three biological replicates. Asterisks indicate a statistically significant difference compared to controls (*P < 0.05; **P < 0.01; ***P < 0.001). **f** Inhibition of C4 cleavage through the classical pathway. Supernatant of haemolysis assays by classical pathway with salivary proteins (15 μg) or without saliva were analysed by Western blot, which were probed with anti-C4 polyclonal antibodies. **g** Action of salivary proteins on C1s enzymatic activity. Assays were carried out by combining purified C4 and C1s to saliva and C4 cleavage was analysed by Western blot. The α (93 kDa), β (75 kDa) and γ (33 kDa) subunits of C4 as well as the α’ fragment (84 kDa) originated from C4 cleavage are indicated by *arrows*. Results of one Western blot out of three are shown in **g** and **f**

To determine which component of the classical complement pathway is affected by *R. microplus* saliva, we first adsorbed human IgG onto a 96-well microplate, then added human serum to allow the deposition of complement components in the activation surface and their recognition by specific antibodies. Saliva was unable to inhibit the deposition of C1q (repeated measures ANOVA: *F*_(4,10)_ = 10.61, *P* = 0.787), the first molecule of the classical complement activation pathway, on the microplate surface (Fig. 2a). However, it inhibited the deposition of C4b (repeated measures ANOVA: *F*_(4,10)_ = 4.77, *P* = 0.029, Fig. 2b) and all the subsequent factors such as C3b (repeated measure ANOVA: *F*_(4,10)_ = 6.86, *P* = 0.011, Fig. 2c), C5b (repeated measures ANOVA: *F*_(4,10)_ = 10.43, *P* = 0.003, Fig. 2d), and C9 (repeated measures ANOVA: *F*_(4,10)_ = 10.78, *P* = 0.003, Fig. 2e).

### *Rhipicephalus microplus* saliva inhibits the cleavage of C4 by the classical pathway

Next, we investigated the mechanism underlying the saliva-induced classical pathway inhibition. Since the inhibitory effect of saliva was first detected on the deposition of C4b, the supernatant of haemolytic assays activated by the classical pathway were evaluated by Western blot with anti-C4 polyclonal antibodies. In the control samples (containing serum, saline and erythrocytes incubated for 30 min), the anti-C4 antibodies recognised the α (93 kDa), β (75 kDa) and γ (33 kDa) subunits of C4 as well as the 84 kDa α’ fragment, which is a product of C4 degradation. There was no α’ fragment in the treatment samples (containing serum, saliva and RBCs incubated for 30 min), indicating inhibition of C4 cleavage (Fig. 2f). As C4 is cleaved by C1s at the classical pathway, we then investigated the action of saliva on C1s. However, the presence of salivary proteins did not affect the C1s-mediated cleavage of C4 (Fig. 2g).

### *Rhipicephalus microplus* saliva inhibits the deposition of C3b and fB by the alternative pathway

**Fig. 3:**
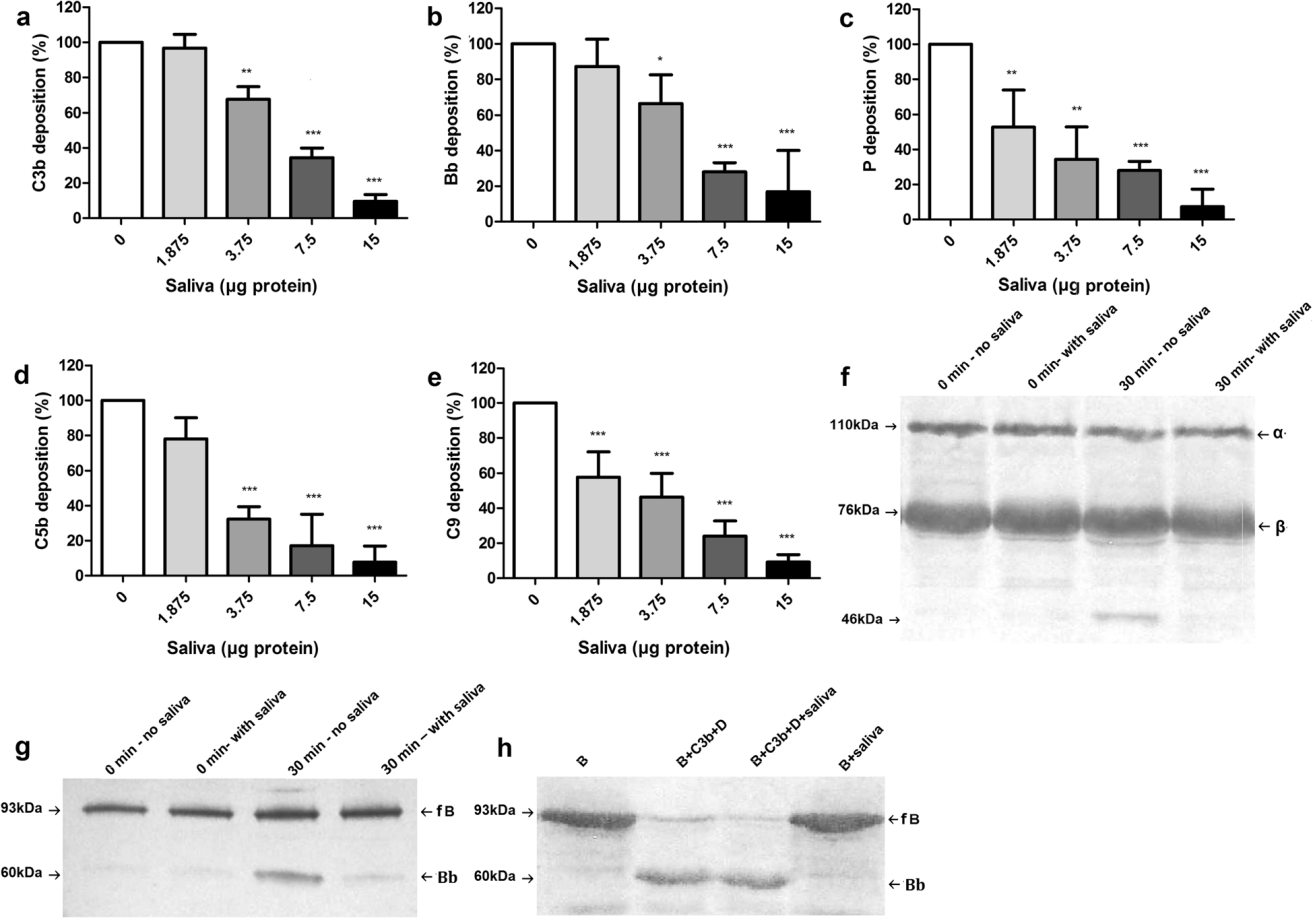
Effect of *Rhipicephalus microplus* saliva on the deposition and cleavage of components of the alternative pathway. **a**-**e** Deposition of C3b, Bb, properdin (P), C5b and C9, respectively, onto the activation surfaces. Bars represent the arithmetic mean ± standard deviation (SD) of three biological replicates. Asterisks indicate statistically significant differences between experimental samples and controls (*P < 0.05; **P < 0.01; ***P < 0.001). **f**, **g** Inhibition of C3 and factor B (fB) cleavage by the alternative pathway, respectively. The supernatant from the haemolysis assay reactions via the alternative pathway or with salivary proteins (15 μg) or without saliva was analysed by Western blot, which was probed with anti-C3 (**f**) or anti-fB (**g**) polyclonal antibodies. **h** Action of saliva on factor D enzymatic activity. Assays were carried out by combining purified fB, C3b and factor D in the absence or presence of salivary proteins (15 μg) followed by Western blot analysis probed with anti-fB polyclonal antibodies. In **f**, the α (110 kDa) and β (76 kDa) subunits of C3 as well as the 46 kDa fragment of the α chain are indicated by *arrows*. In **g** and **h**, fB (93 kDa) and its cleavage product Bb (60 kDa) are indicated by *arrows*. Results of one Western blot out of three are shown in **f**-**h**

To determine which component of the alternative complement cascade is affected by *R. microplus* saliva, we first adsorbed agarose onto the surface of a 96-well microplate, then added human serum to allow the deposition of complement components on the activation surface and their recognition by specific antibodies. Saliva significantly inhibited the deposition of C3b even when small amounts of salivary proteins were used (repeated measures ANOVA: *F*_(4,10)_ = 103.9, *P* < 0.001, Fig. 3a). Consequently, all subsequent factors tested were also significantly inhibited such as Bb (repeated measures ANOVA: *F*_(4,10)_ = 29.26, *P* < 0.001, Fig. 3b) and P (repeated measures ANOVA: *F*_(4,10)_ = 32.12, *P* < 0.001, Fig. 3c), both of which form the C3 convertase and C5b (repeated measures ANOVA: *F*_(4,10)_ = 43.76, *P* < 0.001, Fig. 3d) and C9 (repeated measures ANOVA: *F*_(4,10)_ = 51.07, *P* < 0.001, Fig. 3e), which are MAC components.

### *Rhipicephalus microplus* saliva inhibits the cleavage of C3 and fB through the alternative pathway

The results above show that *R. microplus* saliva acts at the initial stages of activation of the alternative pathway. To further investigate how this inhibition occurs, we evaluated the activity of the saliva on the C3 and fB components. Alternative pathway haemolytic assays were performed and the supernatant of the reactions was evaluated by Western blot using anti-C3 and anti-fB polyclonal antibodies.

In the control samples (containing serum, saline and erythrocytes incubated for 30 min with the activation surface), the anti-C3 antibodies confirmed the presence of the α (110 kDa) and β (76 kDa) subunits as well as a 46 kDa band, which is the degradation product of the C3 α chain, indicating cleavage of C3. The 46 kDa band was not seen in the treatment samples (containing serum, saliva and erythrocytes incubated for 30 min with the activation surface), indicating that saliva inhibits the production of C3b (Fig. 3f). The presence of saliva in the haemolytic assays also prevented the formation of Bb from fB (Fig. 3g), suggesting that the saliva inhibits the cleavage of fB. The production of Bb in an assay where fB was combined to C3b, factor D (D) and saliva indicates that saliva does not inhibit D-mediated cleavage of fB (Fig. 3h).

### *Rhipicephalus microplus* saliva inhibits the binding of fB to C3b through the alternative pathway

To assemble the C3 convertase (C3bBb) of the alternative pathway, fB must bind to C3b and then be cleaved by factor D. Properdin stabilises the binding of C3b to Bb. Therefore, we assessed the effects of *R. microplus* saliva on the formation of the C3b-fB-properdin complex. To this end, purified C3b was adsorbed onto the surface of a 96-well microplate, and then fB and properdin were added to assemble the C3bfBP complex (the precursor of C3 convertase) in the presence of Mg^2+^. Deposition of the components was analysed using anti-fB and anti-properdin polyclonal antibodies.

**Fig. 4:**
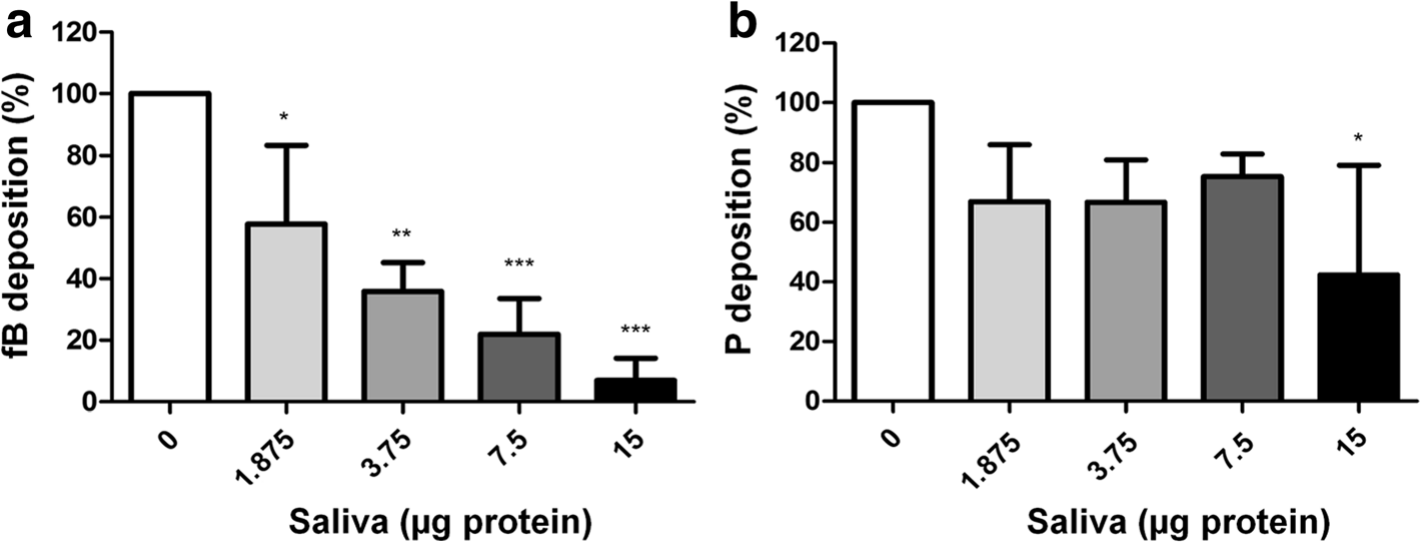
*Rhipicephalus microplus* saliva inhibition upon the binding of factor B and properdin to C3b. fB = factor B (**a**); *P* = properdin (**b**). Assays were performed by adsorbing purified C3b on a 96-well microplate followed by the addition of purified fB and properdin in the presence of Mg^2+^. Different amounts of saliva were tested. The deposition of the components was assessed using anti-fB and anti-properdin polyclonal antibodies. Bars represent the arithmetic mean ± standard deviation (SD) of three biological replicates. Asterisks indicate significant differences from controls (*P < 0.05; **P < 0.01; ***P < 0.001)

Saliva significantly reduced the binding of fB to C3b in a dose-dependent manner (repeated measures ANOVA: *F*_(4,10)_ = 23.26, *P* < 0.001, Fig. 4a), however it was only able to affect the binding of properdin to C3b when 15 μg of salivary proteins were added (repeated measures ANOVA: *F*_(4,10)_ = 3.92, *P* = 0.048, Fig. 4b). This shows that saliva inhibits the alternative pathway by interfering on the association of C3b and fB, thereby preventing the formation of C3 convertase.

### *Rhipicephalus microplus* saliva binds to C3b and properdin but not to factor B to inhibit the alternative pathway

**Fig. 5:**
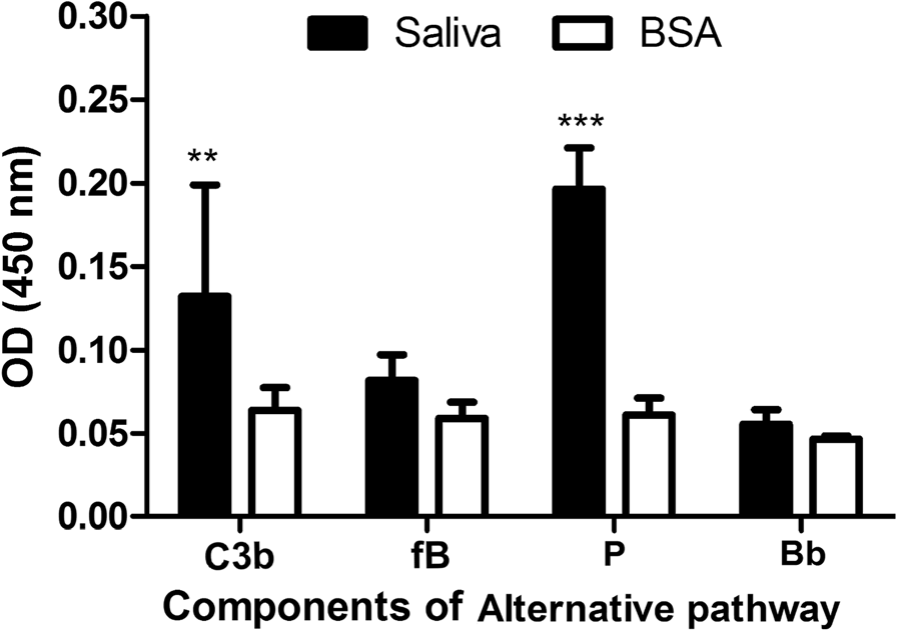
Binding of components from the alternative complement pathway to the saliva of *Rhipicephalus microplus*. fB = factor B; *P* = properdin; BSA = bovine serum albumin (control). Assays were performed by adsorbing saliva (10 μg of salivary proteins) on a 96-well microplate followed by the addition of C3b, fB, properdin and Bb purified components in the presence of Mg^2+^. The binding of the components was assessed using specific polyclonal antibodies. Bars represent the arithmetic mean ± standard deviation (SD) of three biological replicates. Asterisks indicate significant difference from controls (*P < 0.01; ***P < 0.001)

We coated 96-well microplates with saliva and incubated them with complement components (C3b, factor B, Bb and properdin) before adding specific antibodies to detect which components of the complement alternative pathway interact directly with saliva. As shown in Fig. 5, the molecules present in saliva only bound significantly to C3b (Bonferroni, *t* = 3.99, *P* < 0.01) and more strongly to properdin (P) (Bonferroni, *t* = 7.94, *P* < 0.001).

### *Rhipicephalus microplus* saliva does not remove C3 convertase components deposited on alternative pathway activation surfaces

**Fig. 6:**
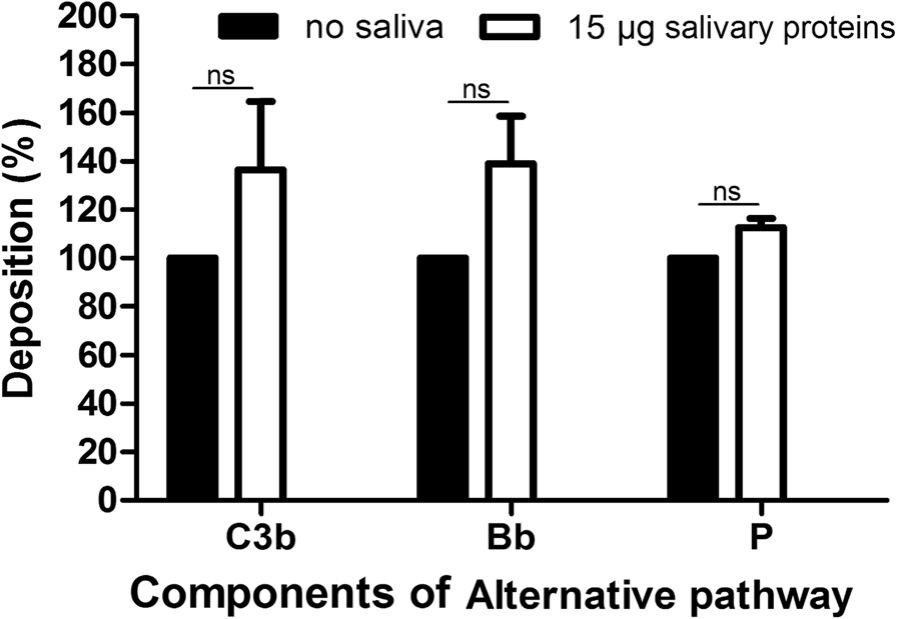
Ability of *Rhipicephalus microplus* saliva to displace complement alternative pathway factors already deposited. Bb = factor Bb; *P* = properdin. Human serum was added to a microplate pre-coated with agarose, leading to the formation of C3 convertase. After 30 min, *R. microplus* saliva was added and the presence of complement component deposits was evaluated using anti-C3, anti-factor B and anti-properdin polyclonal antibodies. Bars represent the arithmetic mean ± standard deviation (SD) from three biological replicates. Statistically significant differences between samples with and without saliva were assessed using *t*-test (C3b *t*_(2)_ = 1.31, *P* = 0.32; Bb *t*_(2)_ = 1.99, *P* = 0.18; P *t*_(2)_ = 3.33, *P* = 0.09). *Abbreviation*: ns, non-significant (*P* > 0.05)

As shown in Figs. 4 and 5, saliva prevents the formation of C3 convertase by inhibiting the deposition of components that are part of this enzyme and the association of fB and C3b. Thus, we investigated whether saliva is also able to displace any of the C3 convertase components (C3b, fB and properdin) deposited via the alternative pathway. To this end, a 96-well microplate previously coated with agarose was incubated for 30 min with human serum before saliva and anti-C3, anti-fB and anti-properdin polyclonal antibodies were added. We found that saliva is not able to remove C3b, fB or properdin once these components of the alternative pathway have been deposited on the activation surface (Fig. 6).

### *Rhipicephalus microplus* saliva does not inhibit deposition of C6-8 or remove pre-deposited components of the MAC

**Fig. 7:**
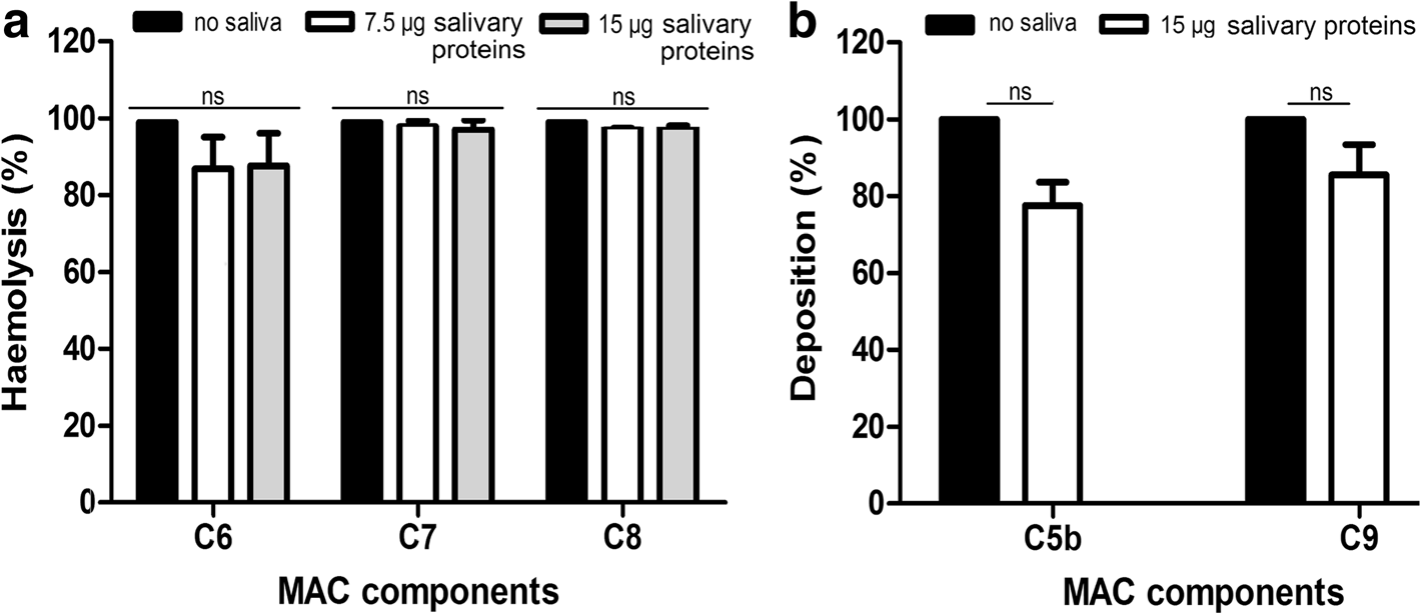
Effect of *Rhipicephalus microplus* saliva on the membrane attack complex (MAC) components’ deposition and removal. **a** Effect on MAC components’ deposition: classical pathway-induced haemolysis was allowed to take place up to the point of C5, C6 or C7 deposition using C6-, C7- or C8-depleted serum, respectively. Normal human serum containing EDTA was then added to the assay to allow the haemolytic process to resume. Haemolysis levels were evaluated in each tube. **b** Effect on MAC components’ removal: alternative complement pathway-induced MAC formation was allowed to occur in agarose-coated 96-well microplates. At this point, saliva was added and the presence of MAC components was evaluated using anti-C5b and anti-C9 polyclonal antibodies. Bars represent the arithmetic mean ± standard deviation (SD) of three biological replicates. **a** Dunnet (C6 *F*_(2,6)_ = 2.32, *P* = 0.21; C7 *F*_(2,6)_ = 0.32, *P* = 0.75; C8 *F*_(2,6)_ = 3.30, *P* = 0.14) and **b** *t*-test (C5b *t*_(2)_ = 3.68, *P* = 0.07; C9 *t*_(2)_ = 1.82, *P* = 0.21). *Abbreviation*: ns, non-significant (*P* > 0.05)

The data presented above revealed that saliva acts at the initial activation steps of the complement cascade. As Jore et al. [17] have shown that saliva inhibits C5b deposition, we investigated whether saliva could also act directly on other components of the final stages of the complement pathway (mainly MAC components). To this end, we performed classical pathway haemolytic assays using sera depleted of one of the following MAC components: C6, C7 or C8. These sera allowed us to investigate the activation of the complement cascade up to the deposition of the C5b, C6 and C7 components, respectively. Normal human serum containing EDTA was then added to the assay to resume the complement cascade without new complement activation, as EDTA chelates Ca^2+^ and Mg^2+^ ions. Inhibition of haemolysis would indicate that saliva could be acting upon each MAC component. However, haemolysis did take place in the treatment samples, suggesting that saliva does not impair the deposition of C6, C7 and C8 on the activation surface (Fig. 7a).

Next, we evaluated whether saliva is also able to displace any of the MAC components (C5b and C9) already deposited on the activation surface via the alternative pathway. To this end, a 96-well microplate coated with agarose was incubated for 30 min with human serum before saliva and anti-C5b and anti-C9 polyclonal antibodies were added. Saliva is not able to remove neither C5b nor C9 after these components of the alternative pathway have already been deposited on the activation surface (Fig. 7b). Indeed, the presence of C9 on the activation surface indicates none of the previous components of MAC were removed.

## Discussion

The huge economic and health impacts in the cattle industry caused by *R. microplus* has fostered research on how these ticks modulate host’s immune system so that, ultimately, therapeutic strategies could be developed against these ectoparasites. It is well known that many tick species use bioactive molecules present in their saliva to inhibit the complement system [8–11]. However, in spite of their importance, only one *R. microplus* salivary protein has been shown to affect the complement cascade [17]. The current study demonstrated that *R. microplus* saliva has other complement inhibitors able to block both the classical and the alternative pathways. Taken together, our results show that ticks have complement inhibitors acting on different points of the cascade, reinforcing the hypothesis that complement inhibition may be extremely important for tick’s success.

The inhibition of both pathways makes the anti-complement activity present in *R. microplus* saliva different from that observed on ticks of the extensively studied genus *Ixodes*. Indeed, most of the *Ixodes* species with complement inhibition activity do so only through the alternative pathway, with both alternative and lectin pathways affected in only one tick species [10].

Saliva of partially and fully-engorged females induced similar levels of haemolysis inhibition when either the classical or alternative pathways were activated. In *I. ricinus*, inhibition of the alternative pathway has been present in the saliva at both engorgement stages although the levels of inhibition observed at the end of feeding were higher than those reported herein for *R. microplus* [43, 44].

The volume and concentration of the salivary proteins were remarkably different between the stages of engorgement. Fully-engorged females produced approximately six times more saliva than partially engorged ones. However, the protein concentration of the saliva produced by the partially engorged females was considerably higher compared to the saliva produced by fully-engorged females. This finding is in accordance to that reported in Tirloni et al. [45]. However, we found that the volume of saliva secreted at both stages of engorgement was approximately 10 times greater than that reported by these authors. These differences in volume of saliva production may be related to the time between the detachment of ticks from the host and pilocarpine stimulation or due to environmental conditions such as temperature and average relative humidity [46].

Here we showed that *R. microplus* saliva blocks the initial steps of the classical pathway. Indeed, saliva did not affect C1q deposition but prevented the deposition of C4b, as confirmed by the data showing that the cleavage of C4 into C4b was blocked. Taken together, these results indicate that inhibition of the classical pathway occurs by preventing the cleavage of C4 by C1s and, hence, the formation of C4b. Saliva was not able to block C1s enzymatic activity. Therefore the possible inhibition mechanism could act preventing C4 binding to the assembled C1, displacing the serine proteases from the C1 complex or directly inhibiting C1r activity. However, further research is required to investigate these hypotheses and improve our understanding on how *R. microplus* saliva inhibits the classical pathway.

To our knowledge, this is the first study showing inhibition of the early steps of the classical complement cascade by the saliva of an ixodid tick. Previous studies on *R. microplus* and other ixodid ticks showed inhibition of the final steps of the cascade [17]. Similar data are only available for other haematophagous arthropods, which also showed saliva-induced inhibition of the classical pathway at the initial stages. Indeed, *Triatoma brasiliensis* saliva prevented the deposition of C4b onto the activation surface [38] and molecules present in *Lutzomyia longipalpis* saliva prevented C4 cleavage and C4b deposition [47].

In haemathophagous animals, inhibition of the classical pathway has been shown to involve a mechanism mediated by calreticulin. In helminths, such as *Necator americanus* and *Haemonchus contortus*, calreticulin sequestrates C1q and prevents its binding to antibodies, thereby blocking the classical pathway [48, 49]. Interestingly, calreticulin is also expressed in the saliva of several tick species [50–52] including *R. microplus* [53]. However, although calreticulin from *R. microplus* saliva has been shown to bind C1q, it did not inhibit the progression of the classical complement pathway [52].

The anticomplement activity described in the saliva of *Ixodes* ticks acts through the alternative pathway by inhibiting C3 convertase formation. This activity is based on the binding of the salivary active molecule to properdin and its removal from C3 convertase. In the absence of properdin, C3 convertase half-life is significantly shortened leading to a reduction in C3 convertase levels, thereby inhibiting the progression of the alternative pathway [15, 16].

The saliva of *R. microplus* also inhibited the haemolysis of rabbit erythrocytes mediated by the alternative pathway. We found that inhibition of the alternative pathway is related to the direct binding of *R. microplus* salivary protein(s) to C3b and properdin. The reduced deposition of C3b on wells covered with agarose was probably a consequence of the lack of formation of C3 convertase. Saliva does not block factor D activity, but it is plausible to suggest that binding of saliva to C3b and to properdin prevents the association of fB to C3b and, to a lesser extent, of properdin to C3 convertase thereby blocking the activation of the alternative pathway.

*Rhipicephalus microplus* saliva did not remove any of the components of C3 convertase, once it was assembled. This is a characteristic of the alternative pathway inhibitory activity found specifically in *R. microplus* saliva because the inhibition promoted by *Ixodes* anti-complement molecules directly removes pre-bound properdin and indirectly removes pre-bound Bb [15, 16]. These results suggest that *R. microplus* salivary anti-complement activity is attributed to a novel molecule with a different mechanism of action from that found in the saliva of the species belonging to the genus *Ixodes*. These data highlight the differences between the salivary activities of blood-sucking arthropods that, in the face of complexity, need to overcome similar anti-tick defence mechanisms of their hosts and ensure feeding success [54]. This hypothesis is supported by analyses of *R. microplus* databases that did not yield any significant hits to known complement inhibitors from *Ixodes* ticks (SALP20, IxACs, IRACs and ISACs).

The action of salivary inhibitors in the early stages of the complement cascade is highly efficient for the ticks: the saliva acts before the amplification of the complement signal which means that considerably fewer salivary molecules will be required to completely block the pathways. The inhibition of C3 convertase assemblage reduces the number of salivary molecules opsonised by C3b and prevents the presentation of these molecules to the host’s immune system [38]. Moreover, without the production of C3b and C5b, inflammatory mediators (such as C3a and C5a) cannot be produced at the bite site, and inflammation can be prevented. Absence of C3a may preclude homeostasis at the bite site since it also induces platelet activation and aggregation [55].

*Rhipicephalus microplus* saliva was previously shown to affect C5b deposition [17]. Here we demonstrated that it has no effect in C6, C7 or C8 deposition and in the removal of C5b or C9, deposited on the activation surface. Inhibition of C5b formation was also observed in argasid ticks for the OMCI, a salivary lipocalin of *O. moubata* [33]. Those molecules bind specifically to C5 thereby preventing its cleavage into C5b and C5a by C5 convertases [17, 32]. Binding to C5 leads to an efficient complement inhibition mechanism because it blocks all activation pathways at once. However, the number of C5 molecules to be inhibited would be considerably high, since the cascade would have been already partially amplified with several inflammatory mediators produced, especially C3a.

## Conclusions

Here we confirmed the presence of anti-complement activity in the saliva of *R. microplus* and showed the mechanisms by which it inhibits the early steps of classical and alternative pathways. Saliva acts strongly at the initial steps of both complement activation pathways by blocking the C4 cleavage and deposition of C4b on the alternative pathway activation surface. In the alternative pathway, saliva acts by binding to initial components of the cascade (C3b and properdin) thereby preventing the C3 convertase formation reducing C3b production and deposition. *Rhipicephalus microplus* saliva is not able to remove C3 convertase components deposited on alternative pathway activation surfaces and has no action on the formation or decay of the C6, C7 and C8 components of the MAC.

We suggest that this inhibitory activity is an important strategy performed by this tick species to modulate host’s immune system. Further research is currently being carried out to identify bioactive agent(s) responsible for the complement inhibitory activity present in *R. microplus* saliva as these molecules may be potential therapeutic targets to reduce the burden of these ectoparasites on cattle farming.

## Abbreviations

BSA, bovine berum albumin; D, factor D; EDTA, ethylenediamine tetra-acetic acid; EGTA, ethylene glycol tetra-acetic acid; fB, factor B; IRAC, *Ixodes ricinus* anticomplement; ISAC, *Ixodes scapularis* anticomplement; IxACs, *Ixodes* anticomplement proteins; MAC, membrane attack complex; MBL, mannose binding lectin; NHS, normal human sera; OmCI, *Ornithodorus moubata* complement inhibitor; P, properdin; PAMPs, pathogen-associated molecular patterns; SALP20, *Ixodes scapularis* salivary protein 20; SDS-PAGE, sodium dodecyl sulfate polyacrylamide gel electrophoresis; TSLPI, tick salivary lectin pathway inhibitor

## Additional file

Additional file 1: Figure S1. Effect of pilocarpine on haemolysis by the classical and alternative pathways. Different concentrations of pilocarpine were tested using normal human serum via the classical (a) and alternative (b) complement pathways, inducing at least 90 % of haemolysis. Bars represent the arithmetic mean ± standard deviation (SD) of three biological replicates. (TIF 384 kb)
