# Supplementary figures and images for: Saliva of Rhipicephalus (Boophilus) microplus (Acari: Ixodidae) inhibits classical and alternative complement pathways
Source: Parasit Vectors. 2016 Aug 11;9:445. doi: 10.1186/s13071-016-1726-8 (PMC4982215; doi:10.1186/s13071-016-1726-8)

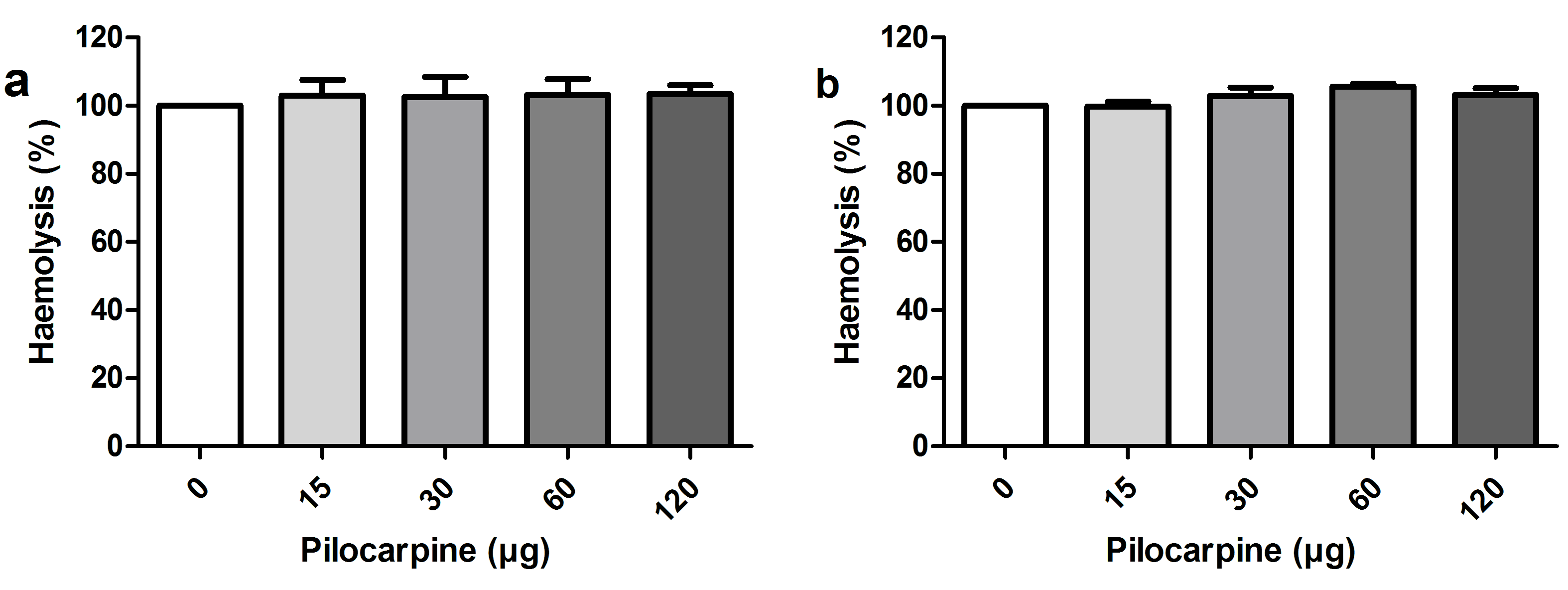

Supplement: Additional file 1: Figure S1. — Effect of pilocarpine on haemolysis by the classical and alternative pathways. Different concentrations of pilocarpine were tested using normal human serum via the classical (a) and alternative (b) complement pathways, inducing at least 90 % of haemolysis. Bars represent the arithmetic mean ± standard deviation (SD) of three biological replicates. (TIF 384 kb) [file 13071_2016_1726_MOESM1_ESM.tif]
